# Submucosal hematoma: a new distinctive sign during emergency upper digestive endoscopy for ammonia ingestion

**DOI:** 10.1186/s12876-018-0809-8

**Published:** 2018-06-20

**Authors:** Moana Gelu-Simeon, Anh-Phuc Chuong, Faouzi Saliba, Guillaume Thiery, Marc Laurent, Claire Vilain, Marius Borel, Leonardo Amaral, Marceline Alexis, Georgette Saint-Georges, Eric Saillard

**Affiliations:** 1 CHU de Pointe-à-Pitre, Service d’Hépato-Gastro-Entérologie, Route de Chauvel, F-97159 Pointe-à-Pitre cedex, Guadeloupe France; 2 INSERM, UMR-S 1085/IRSET, F-35043 Rennes, France; 3 Université Antilles-Guyane, Faculté de médecine Hyacinthe Bastaraud, F-97110 Pointe-à-Pitre Cedex, France; 4 CHU de Saint-Pierre, Service d’Hépato-Gastro-Entérologie, F-97448 Saint-Pierre Cedex, La Réunion France; 5 AP-HP Hôpital Paul Brousse, Centre Hépato-Biliaire, F-94800 Villejuif, France; 6 CHU de Pointe-à-Pitre, Service de Réanimation, F-97139 Pointe-à-Pitre, Guadeloupe France; 7 CHU de Saint-Denis, Service d’Hépato-Gastro-Entérologie, F-97405 Saint-Denis, La Réunion France

**Keywords:** Submucosal hematoma, Aqueous ammonia solution, Ammonium hydroxide, Caustic ingestion, Caribbean island

## Abstract

**Background:**

Submucosal hematoma has never been associated with caustic injuries. Long-term follow-up of patients who ingested ammonia is not well known and ammonia ingestion is rare.

**Methods:**

In a Single-center observational study, prospective data were collected from 2009 to 2013, in patients over the age of 14 years old referred for ammonia ingestion. The emergency and follow-up endoscopic data and the outcome were reported.

**Results:**

Ammonia ingestion occurred in 43 patients. Submucosal hematoma of the gastric wall was a distinctive endoscopic sign observed in 15 (34.8%) cases. Oropharyngeal lesions were present in 30 (69.8%) patients, which was associated with ingestion with suicidal intent in 18 cases. Mild and severe endoscopic lesions (grade IIB to IIIB) were found in 16 (37.2%) cases with 10 (23.3%) cases presenting submucosal hematoma at initial endoscopy. A complete spontaneous gastric healing was frequently observed in 36 (83.7%) cases. In 11 cases with submucosal hematoma, a favourable outcome was observed with a medical treatment, however 6 of these patients had severe endoscopic lesions initially.

**Conclusions:**

Submucosal hematoma of the gastric wall is an endoscopic sign occurring frequently in ammonia ingestion. Submucosal hematoma should be distinguished from necrosis in order to avoid false misclassification in favour of more severe lesions, which would lead to an abusive surgery.

**Electronic supplementary material:**

The online version of this article (10.1186/s12876-018-0809-8) contains supplementary material, which is available to authorized users.

## Background

Suicidal or incidental ingestion of caustic agents still remain a public health issue in many countries and often leads to severe lesions of the oral cavity and the upper gastrointestinal tract (UGT). Among adults, 15,000 cases per year are reported in France and the United states, 90% of which are related to a suicidal attempt [1, 2].

Zargar et al. reported an endoscopic classification of burns, grade 0 to IIIB, depending on the appearance of the mucosa, the depth of ulcers and their extension on the digestive wall and the presence of parietal necrosis [3]. They also demonstrated the prognostic value of early upper endoscopy after caustic ingestion.

Three types of caustic substances are reported worldwide, strong alkalis, strong acids and detergents [4]. The strong alkalis include ammonium hydroxide (called ammonia), potassium hydroxide and sodium hydroxide, which usually cause deep necrosis of the gastric wall and severe and delayed lesions of the UGT [5, 6]. Alkalis are more frequently ingested in western countries such as Asia or India, where injuries from acids are more common [5].

The house cleaning solution which is made of aqueous ammonia, also known as ammonia water or aqua ammonia, is composed of ammonium hydroxide and belongs to the category of strong alkalis (pH = 12); cleaning solutions are generally diluted to a concentration < 7.5% [7, 8]. Several cases of corrosive substance ingestion have been published over the last decade, but there are few data concerning the description of aqueous ammonia solution induced injuries [9–13]. Ammonia ingestion is rare, occurring only in 6.7% of caustic ingestion in a cohort study including 315 patients [9].

Guadeloupe is a French Caribbean Island situated in the lesser Antilles archipelago with 403,000 inhabitants. Ammonia is frequently used for accidental or voluntary ingestion in this region because aqueous ammonia solution is not only considered as a potent cleaning solution, but also in local beliefs, it is thought to drive away evil spirits.

In our experience with ammonia ingestion, we realized that the presence of a hemorrhagic suffusion of the gastric wall, named “submucosal hematoma” (SH), was frequently observed at the initial endoscopic examination. This endoscopic sign has never been described before with caustic ingestion.

We established a prospective observational study with all consecutive patients admitted to our centre with ammonia ingestion outlining their clinical and endoscopic characteristics at admission and outcome. We have evaluated the presence of SH during endoscopic evaluation.

## Methods

### Aim

This study aimed to describe the long-term follow-up of patients that ingested ammonia and report a “new” endoscopic sign named submucosal hematoma detected during emergency esogastro-duodenal endoscopy.

### Study design

This prospective observational study was conducted at the Hepato-Gastroenterology department of the University Hospital of Guadeloupe, from February 2009 to November 2013.

Data were collected and included: the mode of ammonia ingestion as well as its volume, clinical evaluation upon admission and endoscopic esophago-gastric lesions at first endoscopy and at follow-up. The presence of SH following emergency endoscopy was noted separately. The endoscopic description of the esophagus and gastric wall were completed but only the more severe lesions were retained in our analysis. The Ethics Committee of Pointe-à-Pitre Hospital approved this study, and written informed consent was obtained from patients or witnesses in case of unconsciousness upon admission. For patients under 18 years of age, parental consent was obtained.

### Characteristics of participants

All patients 14 years and over, admitted at this hospital for ammonia ingestion were prospectively recruited and had a clinical evaluation by a gastroenterologist.

### Management protocol

Anamnesis of the patients and/or the witnesses tried to determine the time of ingestion, the nature and the quantity of the ingested agents. In case of clinical signs of severity, defined by the presence of hemodynamic shock, mediastinal, subcutaneous emphysema, surgical abdomen or altered consciousness, the patient was intubated, put under mechanical ventilation and a Computed tomography (CT) scan was completed before endoscopy. When a diagnosis for perforation upon admission was done by CT scan, the patient was spared endoscopy and was not included in this study.

After resuscitation and within 6 to 12 h following the caustic ingestion, a senior gastroenterologist performed an endoscopic examination of the UGT to assess the extent of the lesions. Endoscopy was often performed in the presence of the surgeon. In all cases, a gastroenterologist performed the endoscopy and the surgeon was called to attend, under local or general anesthesia and this was regarded as “the initial endoscopy”.

The esophago-gastric lesions were described and classified using *Zargar SA* et al. [3] classification. Grade 0: normal, grade I: edema and hyperemia of the mucosa, grade IIA: superficial and localized ulcerations, grade IIB: circumferential and deep ulcerations, grade IIIA: multiple and deep ulcerations and small scattered areas of necrosis, grade IIIB: extensive necrosis, grade IV: perforation.

In our series, grade I to IIA endoscopic lesions were regarded as “light endoscopic lesions”, and grade IIB to IIIB lesions as “mild and severe endoscopic lesions”.

A follow-up endoscopy at day 8 was completed for patients who had grade IIA to IIIA esophago-gastric lesions [11]. The following endoscopies depended on the evolution of the esophago-gastric lesions at this latter endoscopy. In case of medical management of IIIB lesions, the follow-up endoscopy was proposed at day 21.

### Treatment protocol

According to current guidelines, the management protocol was based on the findings of the initial endoscopic examination of the UGT, but also on clinical signs and symptoms such as abdominal pain [9]. For patients with grade 0 or I esophago-gastric injuries without abdominal pain, no specific treatment was required. Patients were put on a normal diet and were discharged from the hospital within 24 h, after a psychiatric evaluation in the case of suicidal attempts. For patients with grade I injuries with abdominal pain and IIA injuries, oral administration of proton pump inhibitors (PPI) and a normal diet were proposed. For patients with grade IIB or IIIA lesions, the treatment was based on intravenous administration of PPI at 40 mg dosing per day, associated with parenteral nutrition. Regarding the follow-up endoscopy at day 8, oral nutrition was started if the endoscopy showed improvement, or otherwise the endoscopy was repeated at day 21. In such cases, surgical jejunostomy under open laparotomy was performed to allow long-term enteral nutrition. As reported by Cabral et al. [9] in their management algorithm for patients with grade IIIB gastric lesions, an exploratory laparotomy was initially proposed to assess the esophago-gastric wall damage. Ideally, a gastrectomy with esophago-jejunal anastomosis was performed if the necrosis of the gastric wall was confirmed. But in some cases, a gastrectomy combined to an esophagectomy couldn’t be performed due to inexperienced surgeon in esophageal surgery or hemodynamic instability. In a small island, which accounts for 450,000 inhabitants, there is no surgeon experienced in eosophageal surgery and patients must be transferred to a referal center in metropolitan France (7000 km far away). A surgical jejunostomy during exploratory laparotomy was performed for enteral feeding and patients could be transferred for esophagectomy. When the patient was not transportable due to hemodynamic instability, medical management without surgery was performed preferentially.

Patients did not receive systematically antibiotics or corticosteroids. Patients received antibiotics in case of extended necrosis, perforation, or clinical or biological signs of septicemia. In case of stricture development, diet was followed for 6 weeks and a barium swallow test was conducted to assess stricture formation. Endoscopic dilation was the first-line treatment for non-extensive (1 or 2 strictures less than 5 cm) esophageal strictures. Patients who failed endoscopic dilation, defined as stricture persistence after five consecutive sessions during the same year or esophageal perforation during endoscopic dilation, were offered surgical treatment.

### Statistical analyses

Data were collected using an Excel 8.0 file. Statistical analyses were completed with EPI INFO version 3.5.4. Dichotomous data were compared using the χ2 test α = 5%, or Fisher’s exact test when appropriate. Quantitative data were compared using Student’s t test. Exact Poisson confidence intervals were calculated. Data were expressed as median and range; differences at *P* < 0.05 were considered significant.

## Results

Characteristics of the population and clinical presentation at admission.

Between February 2009 and November 2013, 43 patients over the age of 14 years old were admitted for ammonia ingestion at the University Hospital of Guadeloupe. This hospital is the referral hospital of the island for emergency endoscopies and admits the majority of patients having ingested caustic agents. As shown in Table 1, the study included 23 (53.5%) female and 20 (46.5%) male, with median age 49 [15–80] years. The mode of caustic intake couldn’t be documented in one case because the patient was unconscious at admission and no witness was found. Voluntary intake with suicidal intent was most frequent. Oropharyngeal lesions were markedly high in 30 (69.8%) cases and were associated with ingestion with suicidal intent in 18 cases. There was edema in 8/30, desquamation in 6/30 and ulcer in 3/30; no precision about the type of oropharyngeal lesions was made for 13/30 patients.

**Table 1:** Characteristics of the study population and clinical presentation upon admission

	Ammonia (*N* = 43)
Age (median [range], years)	49 [15–80]
Sex female (*n*,%)	23 (53.5)
Intent of ingestion (n,%)	
Accidental Voluntary Unknown	17 (39.5) 25 (58.2) 1 (2.3)
Volume ingested (n,%)	
< 150 ml > 150 ml Unknown	34 (79.0) 7 (16.3) 2 (4.7)
Extradigestive manifestations (n,%)	
Oropharyngeal lesions Clinical signs of severity	30 (69.8) 8 (18.6)
Endoscopic grade (*n*,%)	
0 I IIA IIB IIIA IIIB	9 (20.9) 6 (14.0) 12 (27.9) 6 (14.0) 5 (11.6) 5 (11.6)
Submucosal hematoma (*n*,%)	15 (34.8)

All of the 43 patients underwent emergency upper digestive endoscopy. No endoscopy-related complication was recorded. As shown in Table 1, the results of the initial endoscopy were classified according to the most severe lesions of the esophago-gastric wall. Nine (20.9%) patients were without UGT injuries (grade 0) at initial endoscopic evaluation, they had ingested a small quantity (< 150 ml) of a corrosive agent in all cases and it was accidental intake in 6 cases. No one presented any signs of severity. Eighteen (41.9%) patients had “light endoscopic lesions”, grade I to IIA. They had most frequently ingested a small quantity (< 150 ml) of a corrosive agent in 13. Grade I lesions were more frequently due to accidental intake (4/6 patients) whereas intake due to suicidal intent was more frequent in grade IIB (9/12 cases). The mode of ingestion could not be documented in one patient with altered consciousness at admission. “Mild and severe endoscopic lesions”, grade IIB to IIIB, were found in 16 (37.2%) cases. Only two of them had ingested a volume of ammonia greater than 150 ml but it was frequently with suicidal intent in 11 and extra-digestive signs of severity were present upon admission in 6.

Eight patients presented clinical signs of severity including hemodynamic shock or altered consciousness that required intubation and mechanical ventilation upon admission. Six of them presented SH and severe endoscopic lesions. The two other patients presented grade I endoscopic lesions in one case and grade IIA lesions in the other case. The CT scans showed swelling of the esophageal-wall in one patient with SH, necrosis in one patient without SH. CT scan findings were normal for the 5 other patients with SH and for one patient without SH and grade III B endoscopic lesions.

### “Submucosal hematoma”, a new endoscopic sign

**Fig. 1:**
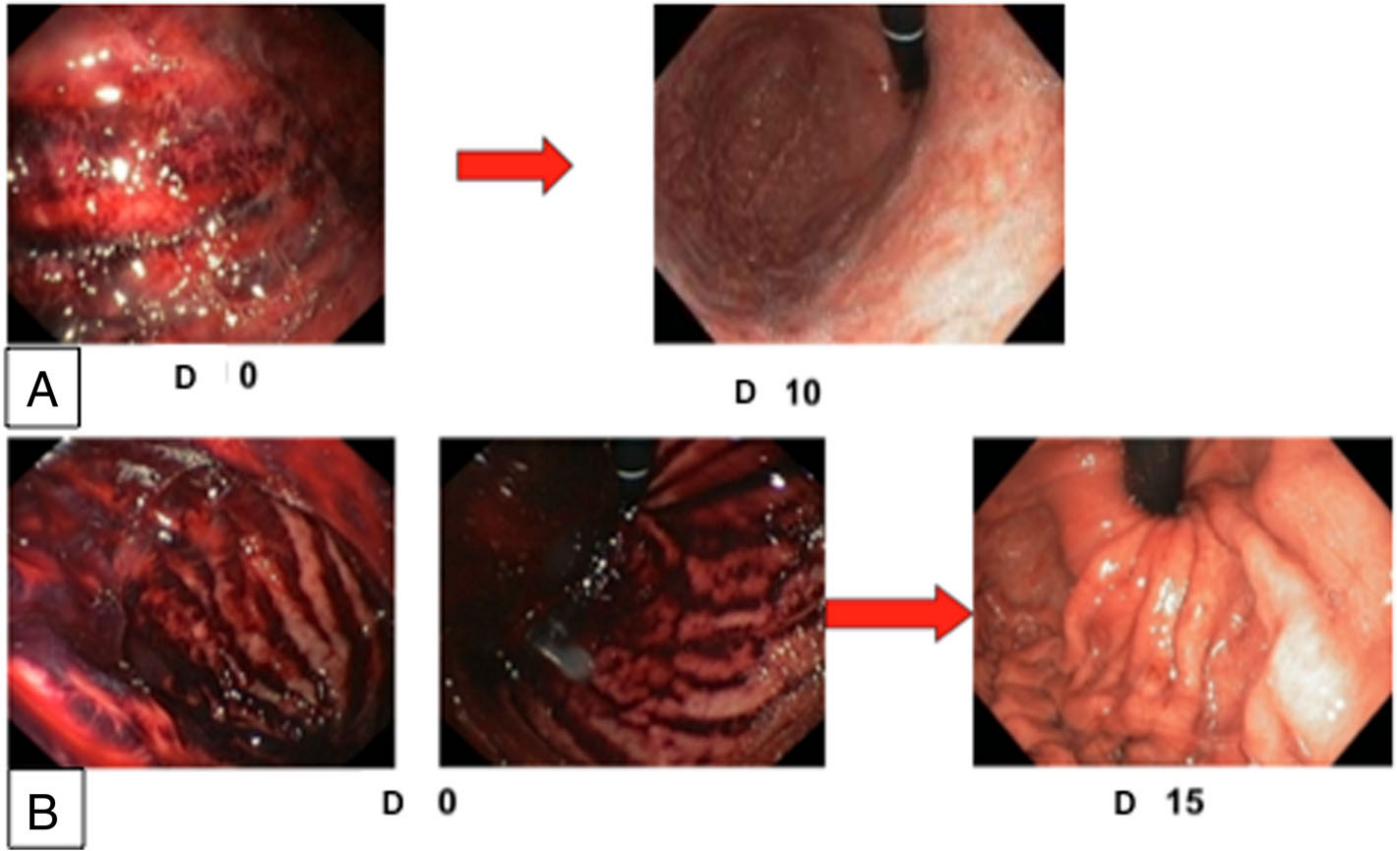
Submucosal hematoma of the fundus gastric wall after ammoniac caustic ingestion. **a**: Endoscopic presentation of case 1 at admission (D0) and day 10 (D10). **b**: Endoscopic presentation of case 2 at admission (D0) en day 15 (D15)

**Fig. 2:**
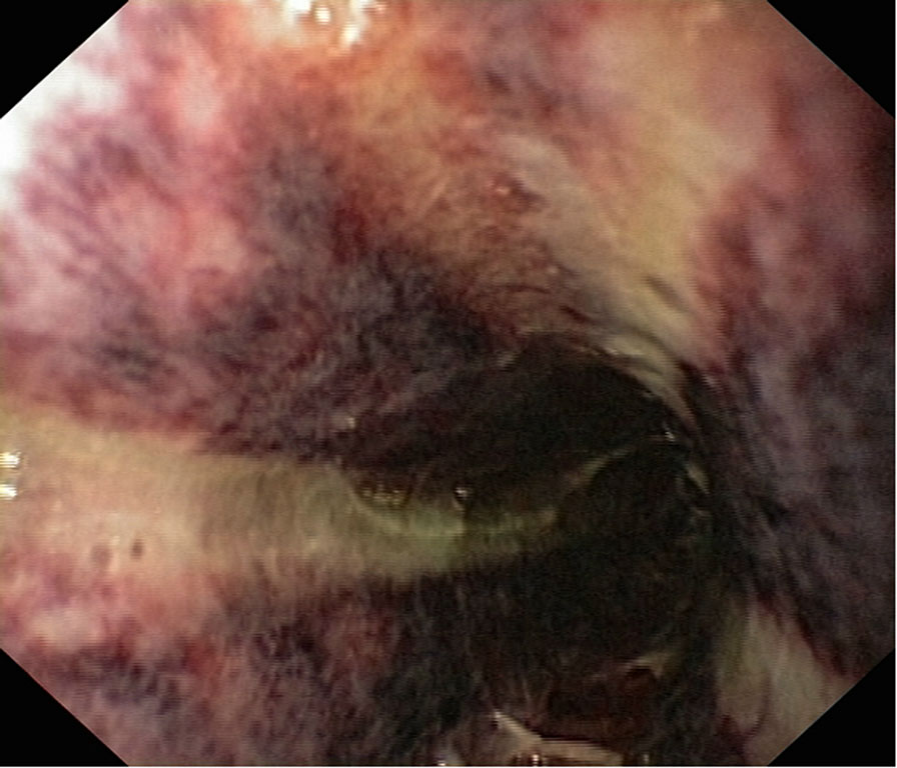
Submucosal hematoma associated with grade IIB of the esophagus wall lesions after ammoniac caustic ingestion

Submucosal hematoma is an endoscopic sign which corresponds to submucosal suffusion hemorrhage on the gastric wall; it can be contrasted with the grayish appearance of necrosis (Fig. 1). SH is preferentially observed in the fundus of the stomach due to the stagnation of the caustic agent in this part of the stomach but it can also affect the esophagus (Fig. 2). It was identified at initial endoscopy in 15 (34.8%) patients in the study. Except for grade 0, SH was reported at each stage of endoscopic grading. It was significantly more frequent in grade IIB or over in 33.3% (grade IIB), 13.3% (grade IIIA) and 20.0% (grade IIIB) respectively; *P* = 0.02 (Table 2). Additionally, SH was much more present in patients who had ingested less than 150 ml of the caustic agent, which concerned the majority of our patients, and didn’t increase with the ingested volume (Table 2). SH occurred more frequently with the presence of clinical signs of severity (*P* = 0.01). The presence of oropharyngeal lesions was particularly high in patients with SH in 12 (80.0%).

**Table 2:** Factors associated with submucosal hematoma and ingestion of ammonia

	Submucosal hematoma present *N* = 15	Submucosal hematoma absent *N* = 28	*P* value
Volume ingested (n,%)			
< 150 ml > 150 ml Unknown	10 (66.7) 3 (20.0) 2 (13.3)	24 (85.7) 4 (14.3) 0	0.16
Endoscopic grade (n,%)			
0 I IIA IIB IIIA IIIB	0 (0) 2 (13.3) 3 (20.0) 5 (33.3) 2 (13.3) 3 (20.0)	9 (32.1) 4 (14.3) 9 (32.1) 1 (3.6) 3 (10.7) 2 (7.1)	***0.02***
Extradigestive manifestations (n,%)			
Oropharyngeal lesions Clinical signs of severity	12 (80.0) 6 (40.0)	18 (64.3) 2 (7.1)	0.2 ***0.01***
Management (n,%)			
Medical Endoscopic dilation Surgical procedures	12 (80.0) 1 (6.7) 2 (13.3)	26 (92.8) 1 (3.6) 1 (3.6)	0.4
Outcome (n,%)			
Favourable Stenosis Death	11 (73.4) 2 (13.3) 2 (13.3)	25 (89.3) 1 (3.6) 2 (7.1)	0.4

Quantitative values are expresssed in n(%). Differences at P<0.05 are bolded

Patients with mild and severe initial endoscopic lesions grade IIB to IIIB (*n* = 16) had SH in 10 cases, and 6 (60%) of these latter had favourable outcome after medical management. The 4 other patients were operated and all died. Six patients had mild and severe lesions without SH, 2 of them died and 1 developed stenosis of the esophagus which was endoscopically treated.

### Evolution of endoscopic lesions and clinical outcome

Twenty-six (60.5%) patients underwent endoscopic follow-up. The median follow-up time for these patients was 8 days [1–21] following admission. The results were a complete healing in 4 patients, a residual inflammation without ulceration in 13, persistent ulcerations in 6, persistent necrosis in 2 and stenosis in 1. Seventeen (39.5%) patients did not undergo endoscopic follow-up, due to initial grade 0 lesions in 9 patients, grade I in 3, and IIA in 2 (including 1 patient who did not return for endoscopic follow-up). One patient with IIB lesions left the hospital prematurely. Two patients with grade IIIB lesions died before the endoscopic follow-up.

During a median endoscopic follow-up of 12 [5–120] days, 19/23 patients with grade IIA to IIIA injuries at admission recovered without sequelae under a conservative management, with or without enteral nutrition. Esophageal strictures occurred in 3 cases; an endoscopic dilation (2 cases) or a surgical reconstruction (1 case) was completed. One (4.3%) patient with grade IIIA lesions died due to sepsis.

In our series, 5 patients had grade IIIB gastric lesions (including 2 with esophageal lesions), with a median endoscopic follow-up of 16 [8–47] days. Surgery was performed immediately in first case and secondarily due to gastric perforation in the other case, but both died. Surgery couldn’t be performed immediately in 3 cases due to the absence of an expert surgeon in esophagectomy. Two of these 3 patients survived with medical management and the last one died.

## Discussion

This prospective study of 43 consecutive patients, included in the University Hospital of Guadeloupe, evaluated caustic injuries induced by ammonia, over a 4-year period. We have reported a new endoscopic sign, SH, which developed in 15 (34.9%) cases. Submucosal hematoma occurred more frequently with mild (grade IIB and IIIA) and severe (IIIB) lesions (*P* = 0.02) and with clinical signs of severity (*P* = 0.01). Oropharyngeal lesions were also particularly high in patients with SH (80.0%).

This is the largest series reported in the literature for ammonia-induced caustic lesions of the UGT. Indeed, Cabral et al. [9] reported 21 (6.8%) cases and Tohda et al. [12] Two (4.1%) cases of ammonia injuries in their series of strong alkalis. In our series, the presentation of ammonia-induced injuries included all types of lesions from 0 to IIIB regardless of the volume ingested or the mode of ingestion. Even if it is composed of ammonium hydroxide, which is a strong alkali agent, ammonia was not significantly associated with more severe endoscopic lesions [6]. Tohda et al. [12] also reported that ammonia usually causes grade I gastric injuries but only 2 patients could be analyzed in their series.

Ammonia has a particular pungent odour as compared to other household alkalis. In this study, we have not observed particular retching or vomiting with this product, however, it could explain the markedly high rate of oropharyngeal lesions seen with ammonia ingestion compared to other caustic agents.

The present data confirm that emergency upper digestive endoscopy remains the cornerstone of the management algorithms for caustic injuries. Here, the endoscopic grading of corrosive injuries at admission was the main factor determining the outcome of the victims of caustic ingestion. In their series, Nunez et al. reported male sex, intentional ingestion, oropharyngeal injuries, presence of clinical symptoms, and the nature of the ingested agent as predictive factors for high-grade injuries [14]. Moreover, the latter factors associated with age, time of exposure, delay before treatment, quantity of ingested substance, and endoscopic grading of injuries of the UGT have been identified as predictive factors for the outcome [10, 15]. In our study, mortality rate was 9.3% and deaths were more frequently correlated to massive and intentional ingestion of ammonia [14].

Submucosal hematoma has been previously described in the literature as an adverse side effect of anticoagulation therapy, but never as a caustic injury complication [16]. It was commonly treated with a medical procedure when present [17]. Tohda et al. [12] described in patients with strong alkalis induced grade II lesions, the endoscopic signs of blisters or frank hemorrhage, contrasting with grey or black coagulation necrosis. In our series, SH was differentiated from necrosis by its hemorrhagic aspect. It was more frequent in mild and severe lesions and associated with the presence of clinical signs of severity upon admission. It appeared from our observations, that there was no correlation between the volume ingested and the extension of lesions in the fundus. However, a medical management was applied to these latter patients with SH and a good outcome was mainly obtained (in 60%). Then, we consider that medical management could be applied to patients presenting SH, particularly when other mild endoscopic lesions were associated. In contrast, in grade IIIB severe injuries, surgical management is usually recommended with a total gastrectomy and blunt, thoracic esophageal stripping in emergency in case of esophageal lesions [18–20]. In our study, 3 patients with grade IIIB lesions were medically managed after exploratory laparotomy with an enteral feeding through jejunostomy. Ultimately, the medical management was chosen due to having inexperienced surgeons in esophageal stripping and the need to transfer the patient with high risk of complications during the long travel-time to metropolitan France [3, 9]. Among these 3 patients who were medically managed, 1 died whereas the 2 others who were surgically managed deceased.

Our study presents some limitations. Noteworthy, CT scans or biopsies were not systematically conducted when SH was present. Indeed, they could be useful to have a better characterization of SH, particularly when associated with necrosis. Moreover, the initial endoscopic staging was mostly completed by one senior endoscopist but was not re-evaluated by another endoscopist in a double-blind exam.

## Conclusions

Ammonia (aqueous ammonium hydroxide solution or aqua ammonia) induced injuries of the upper digestive tract remain a public health issue despite education and regulatory efforts to reduce its occurrence. We identified SH of the gastric wall as an endoscopic sign frequently found in case of ammonia ingestion and mainly observed in mild to severe forms without significant changing of the outcome. SH should be known and recognized by endoscopists in order to avoid misclassification in favour of more severe endoscopic lesions as SH is not easily differentiated from necrosis when severe endoscopic lesions are present for an unaware endoscopist. In this particular case of SH with suspected grade IIIB lesions, a CT-scan should be performed before taking a decision of esophagectomy and/or gastrectomy in order to avoid unjustified surgery.

## Additional file

Additional file 1: All data generated or analyzed during this study are included in the supplementary information files. The collected data were: epidemiological data (age, gender), type of caustic agent (ammonia, volume of the ingested agent, mode of ammonia ingestion), clinical evaluation upon admission (clinical signs of severity, associated oropharyngeal lesions), endoscopic esophago-gastric lesions at first endoscopy (endoscopic lesions according to Zargar classification, presence of submucosal hematoma and its localization), endoscopic follow-up (date, duration and result), therapeutic management (medical, surgical, endoscopic dilation, results of computed tomography). (XLS 70 kb)

## Abbreviations

CT scan: Computed tomography scan
PPI: Proton pump inhibitors
SH: Submucosal hematoma
UGT: Ipper gastrointestinal tract

## References

[1] Lupa M, Magne J, Guarisco JL, Amedee R (2009). Update on the diagnosis and treatment of caustic ingestion. The Ochsner Journal Summer.

[2] Fieux F, Chirica M, Villa A, Losser M-R, Cattan P (2009). Corrosive ingestion in adults. Reanimation.

[3] Zargar SA, Kochhar R, Mehta S, Mehta SK (1991). The role of fiberoptic endoscopy in the management of corrosive ingestion and modified endoscopic classification of burns. Gastrointest Endosc.

[4] Célérier M (2001). Caustic lesions of the esophagus in adults. Ann Chir.

[5] Contini S, Scarpignato C (2013). Caustic injury of the upper gastrointestinal tract: a comprehensive review. World J Gastroenterol.

[6] Zargar SA, Kochhar R, Nagi B, Mehta S, Mehta SK (1992). Ingestion of strong corrosive alkalis: spectrum of injury to upper gastrointestinal tract and natural history. Am J Gastroenterol.

[7] Institut national de recherche et de sécurité pour la prévention des accidents du travail et des maladies professionnelles (2007). Ammoniac et solutions aqueuses. Fiche toxicologique FT16.

[8] Howell JM (1991). Alkalinity of non-industrial cleaning products and the likelihood of producing significant esophageal burns. Am J Emerg Med.

[9] Cabral C, Chirica M, De Chaisemartin C, Gornet J-M, Munoz-Bongrand N, Halimi B (2012). Caustic injuries of the upper digestive tract: a population observational study. Surg Endosc.

[10] Cheng H-T, Cheng C-L, Lin C-H, Tang J-H, Chu Y-Y, Liu N-J (2008). Caustic ingestion in adults: the role of endoscopic classification in predicting outcome. BMC Gastroenterol.

[11] Ertekin C, Alimoglu O, Akyildiz H, Guloglu R, Taviloglu K (2004). The results of caustic ingestions. Hepato-Gastroenterology.

[12] Tohda G, Sugawa C, Gayer C, Chino A, McGuire TW, Lucas CE (2008). Clinical evaluation and management of caustic injury in the upper gastrointestinal tract in 95 adult patients in an urban medical center. Surg Endosc.

[13] Hammond K, Graybill T, Speiss SE, Lu J, Leikin JB (2009). A complicated hospitalization following dilute ammonium chloride ingestion. J Med Toxicol.

[14] Núñez O, González-Asanza C, De la Cruz G, Clemente G, Bañares R, Cos E (2004). Study of predictive factors of severe digestive lesions due to caustics ingestion. Med Clin.

[15] Keh SM, Onyekwelu N, McManus K, McGuigan J (2006). Corrosive injury to upper gastrointestinal tract: Still a major surgical dilemma. World J Gastroenterol.

[16] Jacques M, Desaive C (1986). Submucous hematoma of the cecum under anticoagulant therapy. J Chir.

[17] El-Ashaal YI, Abou-Rebyeh H, Saadeldin YA, Abu-Zidan FM (2011). Submucous traumatic rectal hematoma treated conservatively. J Trauma.

[18] Munoz-Bongrand N, Gornet JM, Sarfati E (2002). Diagnostic and therapeutic management of digestive caustic burns. J Chir.

[19] Cattan P, Munoz-Bongrand N, Berney T, Halimi B, Sarfati E, Celerier M (2000). Extensive abdominal surgery after caustic ingestion. Ann Surg.

[20] Hendrickx L, Hubens A, Van Hee W. Emergency oesophageal stripping, an aggressive approach to acute, necrotic caustic burns of the oesophagus and stomach. Acta Chir 1990;90:46–49. 2356676

